# Telemedicine in the Time of the COVID-19 Pandemic: Results from the First Survey among Italian Pediatric Diabetes Centers

**DOI:** 10.3390/healthcare9070815

**Published:** 2021-06-28

**Authors:** Gianluca Tornese, Riccardo Schiaffini, Enza Mozzillo, Roberto Franceschi, Anna Paola Frongia, Andrea Scaramuzza, on behalf of HCL Expert Pathway Pediatric Group, the Diabetes Study Group of the Italian Society for Pediatric Endocrinology

**Affiliations:** 1 Institute for Maternal and Child Health, IRCCS Burlo Garofolo, 34137 Trieste, Italy; gianluca.tornese@burlo.trieste.it; 2 Diabetes Unit, Bambino Gesù Children’s Hospital, 00165 Rome, Italy; riccardo.schiaffini@opbg.net; 3 Regional Center for Pediatric Diabetes, Section of Pediatrics, Department of Translational Medical Science, University of Naples Federico II, 80131 Naples, Italy; mozzilloenza@gmail.com; 4 Department of Pediatrics, Santa Chiara Hospital Trento, 38122 Trento, Italy; roberto.franceschi@apss.tn.it; 5 Unit of Pediatric Diabetes, Brotzu Hospital, 09134 Cagliari, Italy; annapaolafrongia@aob.it; 6 Division of Pediatrics, ASST Cremona, “Ospedale Maggiore di Cremona”, Viale Concordia 1, 26100 Cremona, Italy

**Keywords:** telemedicine, continuous glucose monitoring, insulin pump, continuous subcutaneous insulin infusion, pediatric diabetes

## Abstract

Background: Use of telemedicine for children and adolescents with type 1 diabetes at the beginning of the COVID-19 pandemic was investigated. Method: 68 Italian pediatric diabetes centers were invited to complete a survey about telemedicine usage in their pediatric patients, allocated to the no-tech group (multiple daily injections and self-monitoring blood glucose) and the tech group (insulin pump and/or flash- or continuous-glucose monitoring). Results: 60.3% of the centers completed the survey. In both the no-tech and tech groups, the most used ways of communication were generic download portals, instant messaging with personal physicians’ mobiles, working emails, and phone calls to physicians’ mobiles, with no difference, except for the use of email being higher in the no-tech group (*p* = 0.03). Seventy-four percent of the centers did not have any systematization and/or reimbursement, with significant differences among regions (*p* = 0.03). Conclusions: Almost all Italian pediatric diabetes centers use telemedicine in a semi-volunteering manner, lacking proper codification, reimbursement system, legal traceability, and accreditation system.

## 1. Introduction

Telemedicine is a term thought up in the 1970s, which literally means “healing at a distance” [1]. It involves the use of information and computer technology to improve patient outcomes by increasing access to care and medical information. Recognizing that there is no definitive description of telemedicine, a 2007 study revealed the existence of 104 peer-reviewed definitions of the word [2] and the World Health Organization adopted a broad delineation of the term: “The delivery of healthcare services, where distance is a critical factor, by all healthcare professionals using information and communication technologies for the exchange of valid information for diagnosis, treatment, prevention of disease and injuries, research, evaluation, and for the continuing education of healthcare providers, all in the interests of advancing the health of individuals and their communities” [3].

Some think that telemedicine differs from telehealth, with the former limited to the service provided only by physicians, and the latter including healthcare professionals in general, such as nurses, pharmacists, and others.

Telemedicine has four key elements: (1) Its purpose is to provide clinical support; (2) it is intended to overcome geographical barriers, connecting users who are not in the same physical location; (3) it involves the use of various types of information and computer technology; (4) its goal is to improve health outcomes [4].

Initially, it was intended to be used, especially in developing countries, to overcome the distances between people and hospitals. However, during these hard times due to COVID-19, where social distancing has become a rule in many countries, including Italy, telemedicine could play a crucial role. In this regard, Hollander and Carr [5] in their recently published perspective on telemedicine stated that “disasters and pandemics pose unique challenges to health care delivery.”

Due to the COVID-19 pandemic, on 9 March 2020, Italy was placed under its first national lockdown. A law decree issued by the Prime Minister’s Office (called #stayhome, or #iorestoacasa in Italian) ordered people across the entire peninsula, with unprecedented measures, to stay at home, and banned all public meetings and travel, excluding only those for “urgent, verifiable work situations and emergencies or health reasons” [6]. This occurrence led Italy to rediscover smart working in many contexts, including telemedicine.

However, if telemedicine already offers a way to be close to patients even from afar, there is still insufficient evidence to support its use in glycemic control and other clinically relevant outcomes among patients with type 1 diabetes [7]. Moreover, there is still little information available about the use of telemedicine in pediatric diabetes, and so far, no studies have evaluated its extension and modalities in Italy.

This survey aimed to investigate in all Italian pediatric diabetes centers at the beginning of the COVID-19 pandemic: (a) The tools used to provide telemedicine services for children and adolescents with type 1 diabetes, both in patients using or not using technological tools (e.g., insulin pumps and/or flash/continuous glucose monitoring systems); (b) the administrative recognition for telemedicine activities; (c) the reimbursement of telemedicine activities.

## 2. Materials and Methods

### 2.1. Participants

All of the 68 Italian pediatric diabetes centers belonging to the Italian Society for Pediatric Endocrinology and Diabetes (ISPED) [8] were invited to complete a survey to collect data about telemedicine usage in their patients.

### 2.2. Questionnaire Development and Pre-Testing

A survey tool was developed composing questions using distinct and interactive steps [9]. The initial list included ten questions evaluated for face and content validity by two expert pediatric diabetologists (G.T. and E.M.) who worked independently and then agreed on the final list, providing feedback on content accuracy, wording, question order, and survey structure. A preliminary version of the survey composed of ten questions was self-administered and piloted in a convenient sample of six pediatric diabetologists. The sample reported that questions were not ambiguous, the wording was straightforward, and the self-administered experience was successful.

According to insulin treatment and blood glucose monitoring, patients were allocated into two groups to detect any differences in telemedicine use: No-tech group for patients using multiple daily injections and self-monitoring blood glucose, and tech group for patients using insulin pumps and/or flash- or continuous-glucose monitoring.

### 2.3. Questionnaire Implementation

A self-administered questionnaire divided into two sections (A and B) was used: In section A, the demographic variables of respondents (i.e., sex and age class) and information about the center (i.e., city, number of individuals with T1DM treatment, setting, and staff) were investigated by one open-ended question (i.e., city) and five closed-ended questions; in section B, data on telemedicine were examined by four closed-ended questions (telemedicine ways used for the no-tech and tech groups, codification, and reimbursement of telemedicine activities). The possible answers, which could be selected through a list of checkboxes shown to the respondents, were decided by the study authors, then modified and confirmed by the authors during the survey structuring phases.

### 2.4. Data Collection Procedure

The survey was web-based, using a commercially available survey host (it.surveymonkey.com, accessed on 10 March 2020). Responses were collected over three weeks, which started on 22 March 2020 up until 12 April 2020. An email reminder was sent two weeks after the initial contact. After ISPED permission, all subscribers of the Diabetes Study Group were contacted by email containing the link to the survey and a brief note outlining the aim of the study, data handling, informed consent statement, invitation to complete the survey, and presentation of the authors. By clicking on the survey link, respondents provided their consent to participate. Participation was voluntary, and no incentives were offered to the participants; all questions were compulsory, although it was possible to quit the questionnaire at any time. The participants were able to review or change their responses using a back button before submitting their answers. Data were downloaded and stored on an encrypted computer, and only the authors had access to the information during all stages of the study. The participants were ensured that their identities would not be disclosed to the investigators: All data were de-identified to maintain confidentiality and data protection [9].

### 2.5. Data Analysis

The empirical analysis was based on the survey data downloaded from SurveyMonkey into Excel spreadsheets and reviewed for accuracy and missing value. Cities were grouped according to geographical regions (i.e., northern, central, and southern Italy). Statistical analysis was conducted using JMP™ software (version 15.1.0, SAS Institute Inc., Cary, NC, USA). Data are presented as frequencies and percentages or as median and interquartile ranges (IQRs). Mann–Whitney rank-sum and two-tailed Fisher exact tests were performed to evaluate the relationship between variables. The Wilcoxon signed-rank test was carried out to check the differences of paired data. A *p*-value < 0.05 was considered statistically significant.

## 3. Results

Among the 68 centers belonging to the Italian Society for Pediatric Endocrinology and Diabetology (ISPED), 41 (60.3%) completed the web-based survey and returned complete data (Table 1). The average time to complete the survey was 3.5 min. In 10 centers, more than one physician completed the survey (two in seven centers and three in three centers) for a total of 54 people who responded to the survey (66.7% female, 45% working in public hospitals, and 55% in academic settings). The percentage of groups divided by age was: 16.4% in the 30–39-year range, 34.6% in the 40–49-year range, 27.3% in the 50–59-years range, and 21.7% in the over 60-year range.

In Table 2, the different methods of using telemedicine have been summarized. The most useful methods to communicate with the diabetes team in the no-tech group were: Generic download portals (e.g., Tidepool, Diasend™, and Glooko™) (80%), instant messaging with personal physicians’ mobiles (76%), working emails (71%), and phone calls to physicians’ mobiles (59%). In the tech group, the ranking of the tools was as follows: Generic download portals (88%), branded download portals (90%), instant messaging with personal physicians’ mobiles (76%), working emails (59%), and phone calls to physicians’ mobiles (59%). There was no significant statistical difference between or within groups, except for the use of email, which was higher in the no-tech group than in the tech group (*p* = 0.03). No significant difference was observed when analyzing the data according to country macro-region (northern, central, or southern), size of the center and hospital, or academic setting (Table 2). Only one center declared not using any tool to communicate with its tech group patients. All of the other centers declared using more than one method to communicate, with a statistical difference between the no-tech group, with a median of 4 (IQR 3–5), and the tech group, with a median of 5 (IQR 4–6) (*p* = 0.002).

In Italy, the health sanitary system is free of charge for all citizens, while the health interventions listed in the “International Statistical Classification of Diseases, Injuries and Causes of Death” (ICD10) are fully or partially reimbursed, according to age, health, and economic status. No telemedicine intervention is officially listed; however, the survey asked if any of the telemedicine interventions have been recognized and reimbursed locally? Most of the centers (74%) did not have any systematization for their telemedicine interventions (Table 3).

The academic centers of central Italy, with less than 100 patients, were those with a higher rate of uncodified service (Table 3). Most centers did not have any reimbursement for telemedicine interventions, with significant differences among regions (100% in southern, 72% in northern, and 47% in central Italy; *p* = 0.03) (Table 4).

## 4. Discussion

On 20 February 2020, the so-called Italian Patient 1 was admitted to the intensive care unit (ICU) of the local hospital due to a deteriorating clinical condition as a result of COVID-19 infection. After a few days, most Italian hospitals, considering the growing number of people infected by COVID-19, decided to suspend outpatient activities. This decision was extended to all hospitals on 9 March 2020, due to the lockdown, which is still ongoing.

For this reason, most ISPED centers have begun telemedicine activities, even if, in many cases, these have never been officially started. Therefore, this survey was conducted, and, to the best of the authors’ knowledge, it is the first to be conducted among the pediatric diabetes centers in Italy and perhaps in Europe.

Telemedicine was originally proposed to facilitate contact between people and healthcare providers in developing countries [1,2,3]. However, to facilitate the containment of the epidemiological emergency due to COVID-19, telemedicine is now the only way to provide healthcare services for the treatment of chronic diseases that do not need physical proximity (e.g., type 1 diabetes in pediatric patients, among others).

The technological development in recent years in the type 1 diabetes field has led to an increase in the use of technology, with the possibility of remote access to continuous glucose monitoring systems and insulin pump data, downloaded by patients in the comfort of their own homes. This opportunity leads to synergy, the involvement of the patients and families, and a sharing of practices that do not require a physical presence (which also remains fundamental in some situations) and could be implemented to save time, travel, and expenses.

In the present survey, all centers, except one, used at least one telemedicine tool, with an average of four methods for the no-tech group and five for the tech group patients, which resulted significantly higher, probably due to the use of insulin pumps and continuous glucose monitoring systems in the latter group.

The most used methods were data download portals, working emails, instant messaging, or phone calls to personal mobiles with no significant differences between the no-tech and tech groups. For the use of working emails only, the no-tech group showed a significantly higher percentage of centers that used them compared to the tech group. The reason could be that the tech group is more prone to using telemonitoring and connection devices than the no-tech group. Indeed, the tech group used the branded download software to a greater extent, which could be of help in data transfer (e.g., CareLink Personal™ and Dexcom Clarity™).

According to the results of this survey, the application of telemedicine appears to be commonly used by Italian pediatric diabetes centers for assisting patients in managing diabetes, as it facilitates the communication of accurate and reliable data between patients and their healthcare providers. It also empowers patients’ attitudes and behavior toward a healthier lifestyle, while providing them with an outlook for better glycemic control. These telemedicine services could be categorized into synchronous (real-time), asynchronous (whereby data are stored and forwarded subsequently), and continuous (remote monitoring).

Nevertheless, it is a shame that only one of four centers reported organization and reimbursement of telemedicine activities. Unfortunately, almost all pediatric diabetes centers in Italy used telemedicine in a semi-volunteering manner because of the lack of proper codification and a reimbursement system. Moreover, most of the methods used (i.e., working emails, text messaging, instant messaging, and phone calls) showed a lack of any legal traceability and are not subject to any accreditation system that might guarantee patients, healthcare providers, and the paying subject [10].

The Italian National Guidelines on Telemedicine published in 2014 [11] state that telemedicine “involves the secure transmission of medical information and data in the form of texts, sounds, images or other forms necessary for the prevention, diagnosis, treatment and subsequent monitoring of patients.” Moreover, it adds that “the use of Information and Communication Technology tools for the treatment of health information or the online sharing of data and health information do not in themselves constitute telemedicine services: as an example, telemedicine does not include health information portals, social networks, forums, newsgroups, emails or others.”

In Italy, though, these guidelines provide the regulatory framework, and the new basic healthcare levels (what the National Health System reimburse) were approved in 2017 [12], comprising of telemedicine as an “alternative and augmentative communication tools and software.” However, the present survey showed that telemedicine in pediatric diabetes is still used in a semi-voluntary way, due to the lack of adequate and uniform platforms, legally accurate traceability of most telemedicine tools, and non-recognition of the work and “televisits” in budgetary terms.

The ability to encrypt emails, thereby ensuring patient confidentiality, is considered difficult when using regular email accounts and none of the respondents reported using certified email accounts. Alternative web-based applications (such as dedicated hospital portals) where the encryption could be implemented would be a good option for security and direct codification, as well as subsequent reimbursement; however, to date, only 11% of centers have had the chance to use this option in Italy.

It is believed that telemedicine must be subjected to an accreditation system that guarantees patients, healthcare providers, and paying subjects, but this system has not yet been implemented.

Although the Italian Health Sanitary System is free of charge (including telemedicine services), the issue of equity problems in telemedicine (similarly to in distance learning programs) should be kept in mind, since poorer families often do not have proper technical devices and a reliable internet connection. Data published in May 2020 by the Italian National Institute of Statistics (ISTAT) revealed that 12.3% of young people aged 6–17 years did not have any personal computer or tablet at home [13].

It is vital to build awareness of these barriers regarding the development of telemedicine and to remove financial barriers (e.g., implementing waivers to purchase essential devices and internet access).

## 5. Conclusions

Almost all of the surveyed Italian pediatric diabetes centers use telemedicine in a semi-volunteering manner, lacking proper codification, reimbursement system, legal traceability, and accreditation system.

Therefore, the time has come, starting from an extraordinary situation, such as the need to assist our pediatric patients during the COVID-19 pandemic, for the Italian National Health System and our hospitals to carefully examine the advantages of telemedicine to fill this gap [14].

## Figures and Tables

**Table 1:** Survey center characteristics.

Center Characteristics	Percentage of Centers	Number of Centers
*Region*		
Northern Italy	43.9%	18
Central Italy	36.6%	15
Southern Italy	19.5%	8
*Number of Individuals with T1DM Treated in the Center*		
<100 individuals	24.4%	10
100–299 individuals	46.3%	19
≥300 individuals	29.3%	12
*Setting*		
Hospital	58.5%	24
Academic	41.5%	17
	Median	IQR
*Staff*		
Pediatric diabetologist	2	(1–2)
Dedicated specialist nurse	1	(1–1)
Dedicated dietician	1	(1–2)
Dedicated psychologist	1	(0–1)

**Table 2:** Telemedicine methods used for the no-tech and tech groups according to the region, the number of patients with type 1 diabetes treated in the center, and the setting. “Others” in no-tech group: (1) Dedicated app on a smartphone; (2) paid consultation platform; (3) Skype/Webex. “Others” in the tech group: (1) “Visitami” app/Zoom; (2) dedicated app on a smartphone; (3) paid consultation platform; (4) Skype/Webex.

		Hospital Dedicated Portal	Generic Data Download Portal	Branded Data Download Portal	Working Emails	Personal Emails	Instant Messaging with Hospital Phone	Instant Messaging with Personal Phone	SMS to Hospital Phone	SMS to Personal Phone	Call to Hospital Phone	Call to Personal Phone	None of the Previous	Other
No-Tech Group	Total	12%	80%		71% *	29%	2%	76%	10%	32%	51%	59%	0%	7%
	Northern	17%	83%		83%	17%	6%	72%	22%	33%	61%	56%		11%
	Central	0%	80%		67%	20%	0%	80%	0%	27%	53%	47%		7%
	Southern	25%	75%		50% §	75%	0%	75%	0%	38%	25%	88%		0%
	*p*					<0.01								
	<100 individuals	10%	90%		60% †	30%	0%	90%	20%	20%	50%	50%		0%
	100–299 individuals	5%	79%		84%	11%	5%	79%	11%	42%	58%	63%		0%
	>300 individuals	25%	75%		58%	58%	0%	58%	0%	25%	42%	58%		25%
	*p*					0.02								0.02
	Hospital	4%	75%		63% ‡	25%	4%	88%	13%	33%	46%	54%		8%
	Academic	24%	88%		82%	35%	0%	59%	6%	29%	59%	65%		6%
	*p*							0.04						
Tech Group	Total	10%	88%	90%	59% *	32%	5%	76%	7%	32%	51%	59%	2%	10%
	Northern	17%	89%	94%	78%	28%	11%	72%	17%	33%	61%	56%	6%	17%
	Central	0%	93%	93%	60%	13%	0%	80%	0%	27%	53%	47%	0%	7%
	Southern	13%	75%	75%	13% §	75%	0%	75%	0%	38%	25%	88%	0%	0%
	*p*				<0.01	<0.01								
	<100 individuals	10%	90%	80%	30% †	40%	10%	90%	10%	20%	50%	50%	10%	0%
	100–299 individuals	5%	84%	89%	79%	21%	5%	79%	11%	42%	58%	63%	0%	5%
	>300 individuals	17%	92%	100%	50%	42%	0%	58%	0%	25%	42%	58%	0%	25%
	*p*				0.03			0.04						
	Hospital	4%	83%	88%	50% ‡	33%	8%	88%	8%	33%	46%	54%	4%	13%
	Academic	18%	94%	94%	71%	29%	0%	59%	6%	29%	59%	65%	0%	6%
	*p*							0.04						
Differences between No-Tech vs. Tech Groups	*p*				* 0.03 § 0.04 † 0.04 ‡ 0.04									

**Table 3:** Codification of telemedicine activities.

	Percentage of Respondents								
		Region			Individuals with T1DM Treated			Practice Setting	
Hospital Parameter for Codification	Total	Northern	Central	Southern	<100	100–299	>300	Hospital	Academic
Methods that should be used	9%	11%	20%	0%	20%	0%	18%	13%	12%
Content of requests from individuals	6%	11%	7%	0%	10%	0%	12%	8%	6%
Time within which the doctor has to reply	2%	6%	0%	0%	0%	0%	6%	0%	6%
Possibility during working hours	7%	11%	7%	0%	0%	7%	12%	4%	12%
Not codified	74%	72%	53%	100%	80%	79%	59%	75%	65%
Other (specify): - Specifying “telemedicine” in the report (*n* = 2) - Codified when using hospital portal (*n* = 2) - With a fee for the individual	12%	6%	27%	0%	0%	11%	25%	8%	18%

**Table 4:** Reimbursement of telemedicine services.

	Percentage of Respondents								
		Region			Individuals with T1DM Treated			Practice Setting	
Hospital Parameter for Reimbursement	Total	Northern	Central	Southern	<100	100–299	>300	Hospital	Academic
Time spent answering	2%	0%	7%	0%	0%	0%	6%	0%	0%
“Exam overview” service	6%	6%	13%	0%	10%	0%	12%	0%	0%
“Diabetes visit” service	26%	28%	47%	0%	10%	29%	41%	0%	0%
None of the above	70%	72% *	47% *	100% *	80%	71%	59%	0%	0%
Other (specify): - After duty hours	2%	0%	7%	0%	0%	7%	0%	0%	0%

* *p* = 0.03, Fisher’s exact test.

## Data Availability

The data are available on request.
